# Supplementary material for: The Role of Empathy in Alcohol Use of Bullying Perpetrators and Victims: Lower Personal Empathic Distress Makes Male Perpetrators of Bullying More Vulnerable to Alcohol Use
Source: Int J Environ Res Public Health. 2023 Jul 3;20(13):6286. doi: 10.3390/ijerph20136286 (PMC10341197; doi:10.3390/ijerph20136286)
Supplement: Supplementary file 1 [file ijerph-20-06286-s001.zip › ijerph-2225345-supplementary.pdf]

The Role of Empathy in Alcohol Use of Bullying Perpetrators and Victims: Lower Personal Empathic Distress Makes Male Perpetrators of Bullying More Vulnerable for Alcohol Use – Supplementary Material

Table S1 – Distribution of bullying roles at baseline (BL) and follow up 1 (FU1) separated by country

			United Kingdom	France	Germany
			N(%)	*N*(%)	*N*(%)
BL	Participants		853	261	1,050
	Bullying role	Perpetrator	21(2.5)	12(4.6)	72(6.9)
		Victim	137(16.1)	22(8.4)	117(11.1)
		Perp.-Victims	36(4.2)	8(3.1)	28(2.7)
		Noninvolved	659(77.3)	219(83.9)	833(79.3)
FU1	Participants		423	153	605
	Bullying role	Perpetrator	7(1.7)	5(3.3)	20(3.3)
		Victim	35(8.3)	3(2.0)	25(4.1)
		Perp.-Victims	5(1.2)	2(1.3)	8(1.3)
		Noninvolved	376(88.9)	143(93.5)	552(91.2)

Item examples for the used questionnaires of bullying, empathy and internalizing and externalizing problems

Bullying: Bullying questionnaire based on Olweus (1986)

Example for bullying perpetration in peer context:

> "I took part in bullying another student/ peer at school"

Example for bullying victimization in peer context:

> "I was bullied at school (a student/ peer said or did nasty or unpleasant things to me)"

Empathy: Interpersonal Reactivity Index (Davis, 1980)

Perspective Taking:

> "I try to look at everybody's side of a disagreement before I make a decision."

Fantasy:

> "When I watch a good movie, I can very easily put myself in the place of a leading character."

Empathic Concern:

> "I often have tender, concerned feelings for people less fortunate than me."

Personal Distress:

"I sometimes feel helpless when I am in the middle of a very emotional situation."

Internalizing and Externalizing Problems: Strengths and Difficulties Questionnaire (SDQ; Goodman, 2001)

Each item is rated on a three-point scale with options "not true", "somewhat true" and "certainly true".

Emotional Problems:

"I worry a lot"

Conduct Problems:

"I fight a lot"

Hyperactivity/Inattention:

"I am constantly fidgeting"

Peer Relationship Problems:

"I am usually on my own"

Prosocial Behavior:

"I try to be nice to other people"

Sex-separated general linear models for the effect of baseline bullying role (perpetrator vs. victim vs. noninvolved) on baseline alcohol use with covariates baseline SDQ subscale "externalizing problems" and baseline SDQ subscale "internalizing problems"

For males, but not females ($p > 0.05$), we observed a significant main effect of BL bullying role on BL alcohol score (see Table S2 and Figure S1). Perpetrators ($M = 1.688$, $SD = 2.148$) had a significantly higher alcohol score than victims ($M = 0.861$, $SD = 1.242$) with p < 0.05 and noninvolved ($M = 1.004$, $SD = 1.427$) with $p < 0.01$. In males and females the covariates SDQ Intern and SDQ Extern reached significance (see Table S2).

Sex-separated general linear models for the effect of follow up 1 bullying role (perpetrator vs. victim vs. noninvolved) on follow up 1 alcohol use and covariates baseline bullying role, baseline alcohol use, SDQ subscale "externalizing problems" and SDQ subscale "internalizing problems" (both measured at baseline and follow up 1)

For males, but not females ($p > 0.05$), we observed a significant main effect of bullying role at FU1 on alcohol score at FU1 (see Table S2 and Figure S1). Perpetrators ($M = 5.240$, $SD = 3.059$) showed significantly higher scores than victims ($M = 2.778$, $SD = 2.636$),

with p < 0.01, and noninvolved (*M* = 3.074, *SD* = 2.506), with $p < 0.001$. In males and females the covariates BL alcohol use, FU1 SDQ Intern and FU1 SDQ Extern reached significance (see Table S2).

Table S2 – Statistical values of univariate general linear models, separated for sex, and with dependent variables baseline alcohol use and follow up 1 alcohol use

		Male				Female			
Dependent variable	Covariates/ *predictor*	*F*	(*df*1,*df*2)	*p*	η^2	*F*	(*df*1,*df*2)	*p*	η^2
BL AUDIT Q×F	BL SDQ Intern	**4.408**	**(1,999)**	**0.036**	**0.004**	**6.427**	**(1,1070)**	**0.011**	**0.006**
	BL SDQ Extern	**26.586**	**(1,999)**	**<0.001**	**0.026**	**72.434**	**(1,1070)**	**<0.001**	**0.063**
	BL BR	**5.366**	**(2,999)**	**0.005**	**0.011**	2.203	(2,1070)	0.111	0.004
FU1 AUDIT Q×F	BL SDQ Intern	0.101	(1,729)	0.750	0.000	0.241	(1,805)	0.623	0.000
	BL SDQ Extern	0.647	(1,729)	0.421	0.001	0.111	(1,805)	0.739	0.000
	BL BR	0.387	(1,729)	0.534	0.001	0.087	(1,805)	0.769	0.000
	BL AUDIT Q×F	**142.198**	**(1,729)**	**<0.001**	**0.163**	**149.991**	**(1,805)**	**<0.001**	**0.157**
	FU1 SDQ Intern	**17.692**	**(1,729)**	**<0.001**	**0.024**	**5.555**	**(1,805)**	**0.019**	**0.007**
	FU1 SDQ Extern	**20.189**	**(1,729)**	**<0.001**	**0.027**	**22.679**	**(1,805)**	**<0.001**	**0.027**
	FU1 BR	**5.395**	**(2,729)**	**0.005**	**0.015**	1.236	(2,805)	0.291	0.003

Note. BL = Baseline, FU1 = Follow Up 1, AUDIT Q×F = AUDIT Quantity × Frequency sub score, SDQ Intern = Strengths and Difficulties Questionnaire subscale “Internalizing Problems”, SDQ Extern = Strengths and Difficulties Questionnaire subscale “Externalizing Problems”, BR = Bullying Role, main predictor of each model is italic, bold marked values are significant at $p < 0.05$ or below.

Figure S1 – AUDIT Quantity × Frequency (Q×F) score separated by measurement point and sex

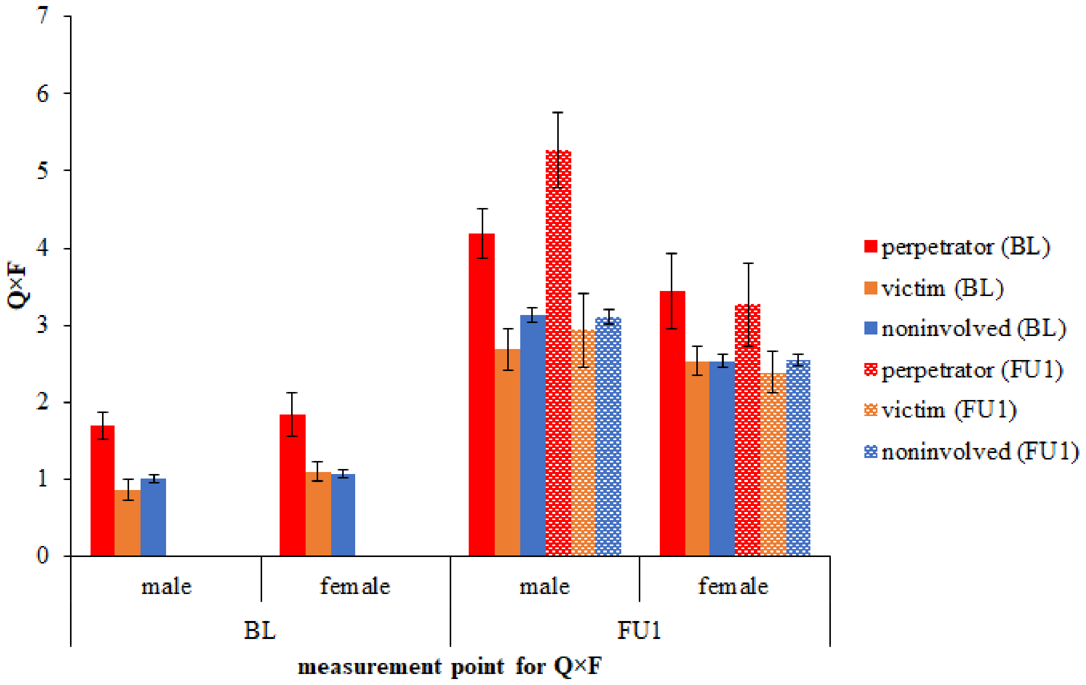

Note. X-axis shows the measurement point of Q×F and y-axis shows Q×F score. Structure of bars represent point of bullying role with BL = baseline and FU1 = follow up 1.

Table S3 – Moderation of the relation between baseline bullying role (predictor) and baseline alcohol use (criterium) by empathy (moderator) for the total sample with covariates sex and SDQ subscales Internalizing Problems and Externalizing Problems

Model	*predictors/* covariates	*F*	*p*	*R²*	*b*	*t*	*p*
PT		**7.820**	**<0.001**	**0.039**			
	constant				1.323	1.723	0.085
	BR				-0.273	-1.307	0.191
	PT				-0.027	-0.564	0.564
	BR × PT				0.009	0.656	0.512
	sex				0.161	1.937	0.053
	SDQ Extern				**0.082**	**5.745**	**<0.001**
	SDQ Intern				**-0.040**	**-2.714**	**0.007**
F		**8.834**	**<0.001**	**0.044**			
	constant				-0.786	-1.067	0.286
	BR				0.340	1.722	0.085
	F				**0.109**	**2.396**	**0.017**
	BR × F				**-0.031**	**-2.504**	**0.012**
	sex				**0.166**	**1.990**	**0.047**
	SDQ Extern				**0.083**	**5.809**	**<0.001**
	SDQ Intern				**-0.042**	**-2.839**	**0.005**
EC		**7.949**	**<0.001**	**0.040**			
	constant				0.843	0.867	0.386
	BR				-0.195	-0.730	0.466
	EC				0.006	0.095	0.924
	BR × EC				0.003	0.205	0.837
	sex				0.162	1.955	0.051
	SDQ Extern				**0.080**	**5.614**	**<0.001**
	SDQ Intern				**-0.043**	**-2.892**	**0.004**
PD		**7.759**	**<0.001**	**0.039**			
	constant				0.680	1.033	0.302
	BR				-0.073	-0.407	0.684
	PD				0.017	0.362	0.718
	BR × PD				-0.005	-0.387	0.699
	sex				0.164	1.946	0.052
	SDQ Extern				**0.082**	**5.715**	**<0.001**
	SDQ Intern				**-0.041**	**-2.728**	**0.006**

Note. "Model" refers to the applied Interpersonal Reactivity Index subscale as moderator, PT = Perspective Taking, F = Fantasy, EC = Empathic Concern, PD = Personal Distress. BR = bullying role at baseline, SDQ Extern = Strengths and Difficulties Questionnaire Subscale "Externalizing Problems", SDQ Intern = Strengths and Difficulties Questionnaire Subscale "Internalizing Problems". Main predictors are written in italic, bold marked values are significant at $p < 0.05$ or below..
